# MSDGFM-YOLO11n-OBB: Selective High-Resolution Difference-Guided RGB–Infrared Fusion for Aerial Oriented Vehicle Detection

**DOI:** 10.3390/s26206634

**Published:** 2026-10-20

**Authors:** Chao Huang, Zhihong Xu

**Affiliations:** School of Computer Science and Technology, Zhejiang University of Science and Technology, 318 Liuhe Road, Liuxia Subdistrict, Xihu District, Hangzhou 310023, China; huangchao@zust.edu.cn

**Keywords:** RGB–infrared fusion, multispectral object detection, oriented object detection, aerial vehicle detection, DroneVehicle, difference-guided fusion, YOLO11-OBB

## Abstract

**Highlights:**

**What are the main findings?**

- MSDGFM combines sample-level RGB/IR allocation with spatially gated cross-modal difference correction and starts exactly from symmetric Mean Fusion.
- Under the unified DroneVehicle Shared-label protocol, P3-only MSDGFM reaches 0.6472 ± 0.0013 mAP@0.50:0.95 over three seeds and outperforms direct Mean Fusion on all three repeated seeds.

**What are the implications of the main findings?**

- Concentrating adaptive fusion at high-resolution P3 while retaining parameter-free P4/P5 fusion provides a compact way to refine localization-sensitive RGB–IR features in aerial OBB detection.
- The 4.069 M-parameter detector exceeds YOLO11s-OBB (IR) by 4.32 percentage points in seed-0 mAP@0.50:0.95 while using 58.1% fewer parameters and 54.0% fewer FLOPs.

**Abstract:**

Aerial vehicle detection using red–green–blue (RGB) and infrared (IR) imagery supports unmanned aerial vehicle (UAV) perception, but oriented localization remains difficult because vehicles are small, dense, arbitrarily oriented, and affected by illumination. Fusion must decide both how cross-modal differences are used and where adaptive fusion is placed. We propose MSDGFM-YOLO11n-OBB, a dual-branch oriented bounding box (OBB) detector with a Modality-Selective Difference-Guided Fusion Module (MSDGFM). Independent backbones preserve modality-specific features. MSDGFM learns sample-dependent RGB/IR weights and injects absolute cross-modal differences through a spatial gate and learnable channel-wise residual; zero initialization makes equal-weight Mean Fusion the exact starting solution. Adaptive fusion is applied only at high-resolution P3, while P4/P5 retain parameter-free Mean Fusion. On DroneVehicle Shared-label data, the three-seed results reach 0.7853 ± 0.0027 mean average precision (mAP)@0.50 and 0.6472 ± 0.0013 mAP@0.50:0.95, exceeding the strongest evaluated single-modal nano-scale OBB baseline by 3.88 and 6.82 percentage points. Relative to matched Mean Fusion, the gain averages 0.83 points in mAP@0.50:0.95 and is positive on all three seeds. In seed-0 capacity control, it exceeds YOLO11s-OBB (IR) by 4.32 points with 58.1% fewer parameters and 54.0% fewer FLOPs. These results support compact, selective difference-guided fusion for aerial RGB–IR OBB detection.

## 1. Introduction

Unmanned aerial vehicle (UAV)-based vehicle detection supports traffic monitoring, urban management, and other aerial perception tasks, but its accuracy is constrained by the visual characteristics of aerial imagery. Vehicles are frequently small, densely arranged, and arbitrarily oriented [1,2,3,4,5], making localization quality particularly important. Oriented bounding boxes (OBBs) are therefore better suited than horizontal boxes for representing vehicle geometry, while illumination and scene variation can further weaken the appearance cues required for precise localization.

Red–green–blue (RGB) and infrared (IR) imagery provide complementary observations: RGB preserves color, texture, and fine appearance, whereas IR can retain target–background contrast when visible-light information becomes less reliable. Recent multispectral detectors have explored attention, modality reweighting, common/differential feature modeling, alignment, and cross-modal interaction [6,7,8,9,10,11,12,13,14,15,16,17,18,19,20]. For aerial OBB detection, two tightly connected design questions remain especially important: how should cross-modal differences be introduced without amplifying uninformative disagreement, and at which pyramid level should adaptive fusion be concentrated so that modality interaction strengthens rather than dilutes localization cues?

These questions are closely coupled. Absolute RGB–IR feature differences can expose complementary responses, but their usefulness varies across samples and spatial locations. At the same time, the high-resolution P3 stage preserves the local contours and layouts that are especially important for oriented vehicles, whereas deeper pyramid stages progressively emphasize semantics. This makes fusion placement a consequential design variable: adaptive interaction should be concentrated where it contributes most to localization and cross-modal complementarity.

We address this problem with MSDGFM-YOLO11n-OBB. Independent RGB and infrared YOLO11n backbones first preserve modality-specific representations. The proposed Modality-Selective Difference-Guided Fusion Module (MSDGFM) then combines two forms of adaptation: a global Softmax branch learns sample-dependent RGB/IR allocation, while a local gate controls where the absolute feature difference contributes to the fused representation. A learnable per-channel coefficient regulates the difference residual. Importantly, the module starts exactly from Mean Fusion because the modality logits and residual coefficient are zero-initialized; the learned behavior, therefore, represents a data-driven refinement of a controlled fusion baseline.

A second design choice is where to apply adaptive fusion. We concentrate MSDGFM at the high-resolution P3 stage, where fine vehicle structure is retained, and use parameter-free Mean Fusion at P4/P5. Validation-set experiments additionally characterize P4-only, P5-only, P3–P4, and P3–P5 alternatives, connecting the selective architecture to its observed placement sensitivity.

The resulting system reaches 0.6472 ± 0.0013 mean average precision (mAP)@0.50:0.95 over three seeds, a 6.82-point gain over the strongest evaluated single-modal nano-scale OBB baseline. More importantly for isolating the fusion mechanism, MSDGFM P3 also improves over direct RGB–IR Mean Fusion on every repeated seed. The benefit is distributed across all five vehicle categories, with particularly strong gains for van and truck. A capacity-control comparison further shows that the proposed 4.069 M-parameter model surpasses YOLO11s-OBB (IR) by 4.32 points in seed-0 mAP@0.50:0.95 while using substantially fewer parameters and floating-point operations (FLOPs).

The main contributions are:

1. Controlled global-local difference-guided fusion. MSDGFM combines sample-level RGB/IR allocation with spatially varying absolute-difference injection. Zero-initialized modality logits and residual coefficients embed symmetric Mean Fusion as the exact starting solution, after which sample-dependent allocation and gated difference correction are learned from data.

2. Placement-controlled selective fusion for aerial OBB detection. We evaluate five single- and multi-level placements on the validation split and focus the final design on MSDGFM at P3, with parameter-free Mean Fusion at P4/P5. This concentrates learned cross-modal refinement at a localization-sensitive resolution while keeping the deeper fusion path simple.

3. Strong, repeatable accuracy–capacity evidence. Across three seeds, the final model reaches 0.6472 ± 0.0013 mAP@0.50:0.95, improves direct RGB–IR Mean Fusion on every repeated seed, and improves all five vehicle categories. In the seed-0 capacity control, it exceeds YOLO11s-OBB (IR) by 4.32 percentage points while using 58.1% fewer parameters and 54.0% fewer FLOPs.

## 2. Related Work

### 2.1. Oriented Object Detection in Aerial Imagery

Aerial object detection differs from conventional ground-view detection because objects frequently exhibit arbitrary orientations, large scale variations, dense spatial distributions, and complex backgrounds. The DOTA benchmark formalized many of these challenges by providing large-scale aerial images with oriented annotations, and it has subsequently become a standard benchmark for rotated object detection [1]. In such scenes, horizontal bounding boxes often include excessive background or overlap strongly with neighboring instances, motivating detectors that explicitly model object orientation.

Early oriented detectors largely extended two-stage detection pipelines. RoI Transformer learns to transform horizontal RoIs into rotated RoIs and extracts orientation-aware features for subsequent classification and regression [2]. ReDet further incorporates rotation-equivariant representations and rotation-invariant RoI alignment to encode orientation changes more explicitly [3]. Oriented R-CNN simplifies the two-stage formulation by directly generating high-quality oriented proposals and provides a strong accuracy–efficiency baseline for rotated detection [4]. Oriented RepPoints instead represents oriented objects using adaptive point sets [5].

These detectors establish strong geometric modeling for aerial imagery, but they are primarily single-modal. Our work keeps the downstream OBB formulation intact and instead focuses on the RGB–IR representation supplied to the detector.

### 2.2. RGB–IR Multispectral Object Detection

RGB–IR detection exploits the complementary sensing characteristics of visible and infrared imagery. RGB images generally retain richer appearance and texture information, whereas infrared images can remain informative when visible-light contrast deteriorates. The central challenge is therefore not merely to combine two inputs, but to determine how much information should be taken from each modality and how cross-modal redundancy or disagreement should be handled.

A substantial body of multispectral detection research has focused on adaptive feature fusion. MBNet identified modality imbalance as an important optimization issue and introduced differential-modality-aware fusion together with illumination-aware feature alignment [6]. Guided Attentive Feature Fusion (GAFF) learned attention-based multispectral feature integration for RGB–thermal pedestrian detection [7]. CMAFF explicitly distinguished common-modality and differential-modality information and used separate attention mechanisms to enhance modality-specific features while selecting shared information [8]. These works show that treating the two modalities identically is often suboptimal.

Transformer-based methods subsequently strengthened long-range cross-modal interaction. Cross-Modality Fusion Transformer (CFT) introduced transformer-based attention to model intra-modal and inter-modal dependencies [9], while ICAFusion employed iterative cross-attention to progressively exchange information between modalities [10]. C^2^Former further introduced calibrated and complementary transformer interaction for RGB–infrared detection [20]. More recent approaches continue to investigate modality-aware and scene-adaptive fusion. RGB-X Object Detection via Scene-Specific Fusion Modules uses scene-dependent fusion modules for heterogeneous RGB-X inputs [11], while recent WACV work explicitly addresses cross-modal information complementarity and modality imbalance [12]. WaveMamba explores frequency-aware RGB–IR interaction using wavelet decomposition and Mamba-style sequence modeling [13].

Recent studies published in Sensors in 2026 further sharpen this design space. GEM-YOLO uses global-context gating for scene-dependent RGB/thermal weighting [21], GMD-YOLO combines gradient-aware fusion with a broader YOLO11 redesign [22], and MDCFT-HSM controls auxiliary-modality injection through a protected branch and zero-initialized residual mappings [23]. Together with earlier adaptive and Transformer-based methods, these studies establish modality reliability, complementary feature modeling, and controlled cross-modal interaction as central design dimensions of RGB–IR detection.

### 2.3. RGB–IR Aerial Vehicle Detection

RGB–IR aerial imagery introduces additional difficulties beyond conventional multispectral pedestrian or ground-view detection. DroneVehicle established a large-scale paired RGB–infrared aerial vehicle benchmark and uncertainty-aware cross-modal detection across diverse urban scenes [14]. TSRA subsequently highlighted translation, scale, and rotation discrepancies between paired modalities and introduced explicit feature calibration [15], making alignment and localization particularly relevant to aerial oriented detection.

One line of recent work emphasizes adaptive modality utilization and reliability-aware fusion. DGE-YOLO adopts dual-stream extraction and structured feature aggregation on DroneVehicle [16]; GEM-YOLO and MDCFT-HSM further explore scene-dependent gating and controlled auxiliary-modality injection [21,23]. Recent UAV RGB–IR studies further explore gated recalibration and bidirectional cross-modal attention [24], alongside adaptive multimodal feature integration [25]. These methods show the value of adapting cross-modal contribution to the sensing condition and detector representation.

A second line organizes cross-modal interaction across the feature pyramid. TFFB applies Cross-Transformer fusion at P3, P4, and P5 [26], MCF-YOLO combines high-resolution and deeper hierarchical interaction [27], and AMSRDet uses global fusion together with adaptive multi-scale weighting on DroneVehicle [28]. DVIF-Net concentrates cross-modal fusion at P4 and complements it with edge-information enhancement for UAV small-target detection [29]. These studies make fusion level an explicit architectural consideration for UAV small-object perception.

A third line focuses on discrepancy, complementarity, and interference-aware modeling. DMM introduces disparity-aware fusion for multispectral oriented detection [19], while MO R-CNN brings heterogeneous multimodal representation learning into an OBB framework [18]. SIM-PCSR performs selective interaction and complementary refinement around the P3 key layer [30]. Noise-aware approaches further suppress modality-specific interference before or during fusion, including purification-before-fusion [31] and attention-based suppression of cross-modal interference [24]. Cross-modal edge enhancement provides another complementary-detail perspective under adverse UAV conditions [17].

Together, recent aerial RGB–IR studies move the field toward adaptive modality utilization, discrepancy-aware complementary modeling, and level-aware interactions [14,15,16,17,18,19,20,21,22,23,24,25,26,27,28,29,30,31]. This progression motivates a focused question for localization-sensitive aerial OBB detection: how can a stable fusion baseline be refined by sample-dependent modality allocation and local difference guidance, and at which pyramid level should that adaptive interaction be concentrated?

### 2.4. Positioning of the Proposed Method

Recent multispectral detectors provide complementary solutions to modality reliability, alignment, discrepancy modeling, and multi-level interaction [14,15,16,17,18,19,20,21,22,23,24,25,26,27,28,29,30,31]. Table 1 summarizes the representative design space that motivates the two controlled axes studied here: fusion mechanism and fusion placement.

MSDGFM combines sample-dependent two-way Softmax RGB/IR allocation with a spatial gate on the absolute cross-modal difference and a zero-initialized channel-wise residual, starting from exact Mean Fusion. This differs from CMAFF [8], which uses common- and differential-modality attentions for feature selection and enhancement, and GAFF [7], which employs intra- and inter-modality attentions instead of an explicit difference residual. MDCFT-HSM [23] protects a selected modality stream and injects auxiliary information through zero-initialized residuals at multiple stages; our two streams contribute symmetrically at initialization, with adaptive fusion concentrated at P3 and Mean Fusion at P4/P5. Fixed-P3 global/local ablations and a same-framework CMAFF-style adaptation assess this particular fusion interface.

## 3. Proposed Method

### 3.1. Overall Architecture

**Figure 1:**
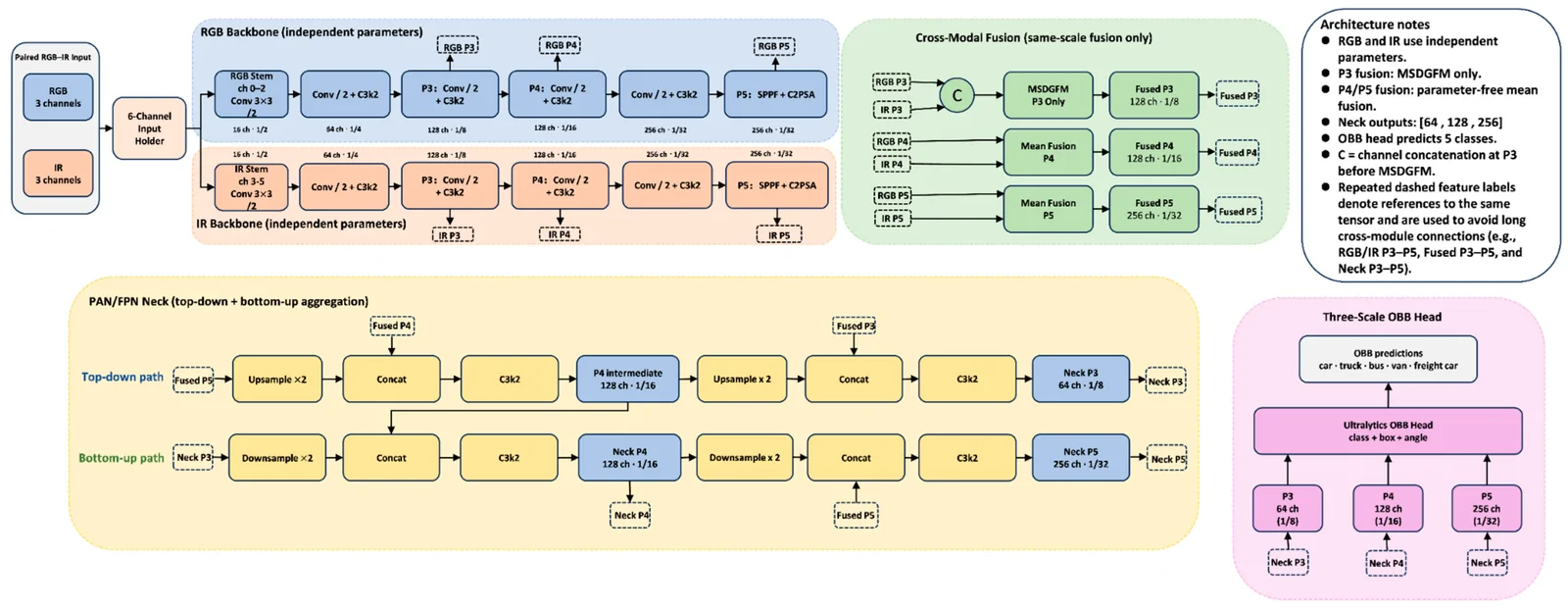
Overall architecture of the final MSDGFM-YOLO11n-OBB (P3) detector. Red–green–blue (RGB) and infrared (IR) images are processed by independent YOLO11n backbones. The Modality-Selective Difference-Guided Fusion Module (MSDGFM) is used only at P3, while P4 and P5 use Mean Fusion before path aggregation network/feature pyramid network (PAN/FPN) aggregation and three-scale oriented bounding box (OBB) prediction. In the schematic, blue and orange denote the RGB and IR backbone branches, light green denotes cross-modal fusion, pale yellow denotes the PAN/FPN neck, and pink denotes the OBB head; arrows indicate the direction of feature flow.

MSDGFM-YOLO11n-OBB adopts a dual-branch YOLO11n-OBB architecture with selective pyramid fusion, as illustrated in Figure 1. Given a paired RGB–IR sample, the RGB image and the infrared image are packed into a six-channel tensor and routed to two independent feature-extraction branches. The two branches use the same backbone topology but maintain independent parameters, preserving modality-specific representations before fusion.

Let the paired input be

**X** = [**I***_r_*,**I***_i_*] ∈ ℝ*^B^*^×6×*H*×*W*^, (1)

where **I***_r_* ∈ ℝ*^B^*^×3×*H*×*W*^ and **I***_i_* ∈ ℝ*^B^*^×3×*H*×*W*^ denote the RGB and IR images, respectively. The implemented input holder preserves the six-channel tensor, while the two independent stem modules explicitly select channels 0:3 and 3:6 for the RGB and IR branches.

In Equation (1), *B* denotes the batch size, while *H* and *W* denote the input image height and width. At later pyramid levels, *H* and *W* denote the spatial dimensions of the corresponding feature map.

Each branch follows the YOLO11n backbone topology, composed of convolutional downsampling, C3k2 blocks, SPPF, and C2PSA. Three pyramid features are extracted from each branch:

(2) $$\{F_{r}^{3},F_{r}^{4},F_{r}^{5}\},\;\{F_{i}^{3},F_{i}^{4},F_{i}^{5}\},$$

corresponding to spatial strides of 8, 16, and 32, respectively. In the final nano-scale implementation, the P3 and P4 backbone features contain 128 channels, whereas P5 contains 256 channels.

The key design of the proposed detector is selective cross-modal fusion. At P3, the paired RGB and IR features are concatenated along the channel dimension and processed by the proposed Modality-Selective Difference-Guided Fusion Module (MSDGFM). At P4 and P5, parameter-free Mean Fusion is retained. The resulting fused pyramid features are then passed to the standard YOLO11 path aggregation network/feature pyramid network (PAN/FPN) neck. After top-down and bottom-up aggregation, the neck outputs three feature maps with 64, 128, and 256 channels, which are forwarded to the oriented bounding box head for five vehicle categories.

In the name MSDGFM, Modality-Selective refers to the learned sample-dependent RGB/IR allocation, whereas Difference-Guided refers to the locally gated absolute-difference residual. The module itself operates on one paired feature level at a time. The final detector instantiates it only at P3; pyramid placement is therefore an independently evaluated architectural choice rather than an internal multi-scale operation of the module.

### 3.2. Dual-Branch RGB–IR Feature Extraction

The RGB and IR streams therefore use structurally identical but independently parameterized YOLO11n backbones, with cross-modal interaction delayed until pyramid features are available. This separation keeps modality-specific feature learning distinct from the subsequent controlled fusion stage.

For pyramid level *l* ∈ {3,4,5}, the modality-specific features are expressed as

(3) $$F_{r}^{l}=B_{r}^{l}\left(I_{r}\right),\;F_{i}^{l}=B_{i}^{l}\left(I_{i}\right),$$

where $B_{r}^{l}\left(\cdot \right)$ and $B_{i}^{l}\left(\cdot \right)$ denote the RGB and IR feature extractors up to pyramid level *l*. The two functions share the same architecture but not the same parameters.

The independently extracted features are fused only after modality-specific representation learning. For P3, the two 128-channel features are concatenated into a 256-channel tensor and fed to MSDGFM, which returns a 128-channel fused feature. For P4, two 128-channel features are fused into a 128-channel representation through Mean Fusion, and for P5, two 256-channel features are fused into a 256-channel representation. This design preserves the feature dimensions expected by the subsequent PAN/FPN neck.

### 3.3. Modality-Selective Difference-Guided Fusion Module (MSDGFM)

#### 3.3.1. Feature Projection and Explicit Difference Representation

**Figure 2:**
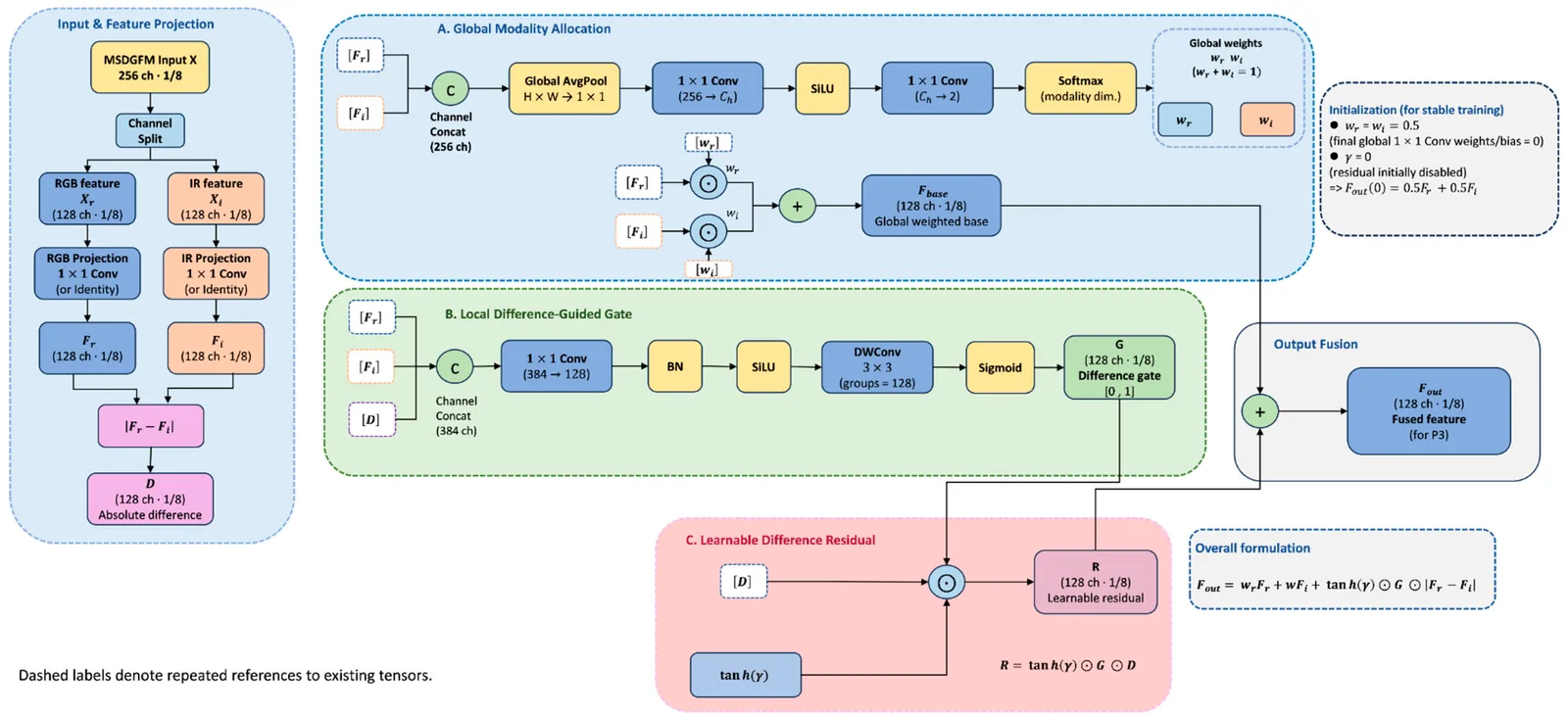
Exact data flow of MSDGFM. The module combines global Softmax modality allocation with a local difference-guided gate and a learnable per-channel residual coefficient. Blue/orange feature blocks distinguish the RGB and IR pathways; the light-blue, light-green, and light-pink regions represent global modality allocation, local difference gating, and learnable difference residuals, respectively. Arrows show data flow and circular symbols indicate the feature operations labeled in the diagram.

Fixed averaging assumes that RGB and IR are equally informative for every sample, whereas directly injecting all cross-modal differences can also amplify uninformative disagreement. MSDGFM separates these two decisions: a global branch learns sample-dependent modality allocation, while a locally gated residual determines where explicit cross-modal difference should contribute. The structure is shown in Figure 2. Consider an input obtained by channel-wise concatenation of two modality features. The input is first divided into equal RGB and IR halves, denoted X_r and X_i. Channel projection is performed as.

For clarity, C denotes the channel dimension of the projected features, H and W are spatial dimensions, r is the global MLP reduction ratio (16 in the implementation), GAP denotes global average pooling, BN denotes batch normalization, σ denotes the logistic sigmoid, and ⊙ denotes element-wise multiplication. The local depthwise convolution uses C groups. The channel-wise residual coefficient γ is trained jointly with all model parameters and is transformed by tanh before use.

**F***_r_* = *ϕ_r_*(**X***_r_*),  **F***_i_* = *ϕ_i_*(**X***_i_*), (4)

where *ϕ_r_*(⋅) and *ϕ_i_*(⋅) are modality-specific 1 × 1 projections when the input and output channel dimensions differ; otherwise, they reduce to identity mappings. In the final P3 configuration, the input half already contains 128 channels and the module output is also 128 channels, so both projections become identity mappings.

MSDGFM explicitly constructs the cross-modal difference tensor

**D** = |**F***_r_* − **F***_i_*|. (5)

The absolute difference captures the magnitude of modality disagreement without assigning a fixed directional meaning to RGB-minus-IR or IR-minus-RGB responses.

**Difference-form interpretation.** The implemented unsigned absolute difference is invariant to swapping the two input modalities and encodes the magnitude rather than the direction of disagreement. A signed RGB-minus-IR difference instead retains modality order and may change sign upon swapping modalities; a squared difference would emphasize large discrepancies more strongly. These distinctions are analytical; no separate signed- or squared-difference experiments are included.

#### 3.3.2. Global Modality Allocation

The first branch of MSDGFM learns scene-dependent global modality weights. The projected RGB and IR features are concatenated and globally pooled:

**z** = GAP([**F***_r_*;**F***_i_*]). (6)

Here, GAP denotes global average pooling. The pooled descriptor is processed by two 1 × 1 convolutions with a SiLU activation:

**a** = **W**_2_(*δ*(**W**_1_(**z**))), (7)

where *δ*(⋅) denotes SiLU. The hidden dimension is

The trainable matrices *W*_1_ and *W*_2_ are implemented as consecutive 1 × 1 convolutions; *W*_1_ reduces the pooled channel descriptor to *C_h_* channels, and *W*_2_ predicts two modality logits prior to Softmax.

(8) $$C_{h}=\max\left(\frac{C}{r},8\right),$$

with reduction ratio *r* = 16. A softmax operation along the modality dimension gives

[*w_r_*,*w_i_*] = Softmax(**a**),  *w_r_* + *w_i_* = 1. (9)

The globally weighted base feature is

**F***_base_* = *w_r_***F***_r_* + *w_i_***F***_i_*. (10)

#### 3.3.3. Local Difference-Guided Gate

Global weighting captures sample-level modality allocation. To model spatially varying disagreement, MSDGFM complements it with a second branch driven by the explicit difference tensor. The RGB feature, IR feature, and absolute-difference feature are concatenated:

**U** = [**F***_r_*;**F***_i_*;**D**] ∈ ℝ*^B^*^×3*C*×*H*×*W*^. (11)

The local gate is computed as

**G** = *σ*(DWConv_3×3_(*δ*(BN(Conv_1×1_(**U**))))), (12)

where *σ*(⋅) is the sigmoid function and DWConv_3×3_ is a depthwise convolution with groups equal to the number of channels, and BN denotes batch normalization. The resulting gate satisfies

**G** ∈ [0, 1]*^B^*^×*C*×*H*×*W*^. (13)

#### 3.3.4. Learnable Difference Residual

To control the strength of the difference pathway, MSDGFM introduces a learnable per-channel parameter

**γ** ∈ ℝ^1×*C*×1×1^. (14)

The difference-guided residual is

**R** = tanh(**γ**)⊙**G**⊙**D**, (15)

and the final fused representation is

**F***_out_* = *w_r_***F***_r_* + *w_i_***F***_i_* + tanh(**γ**)⊙**G**⊙|**F***_r_* − **F***_i_*| (16)

where ⊙ denotes element-wise multiplication.

The implementation uses a controlled Mean Fusion initialization. The weights and bias of the final global weighting layer are initialized to zero, so the two initial softmax coefficients are exactly 0.5. Meanwhile, **γ** is initialized to zero, so the difference-guided residual is initially disabled. Therefore,

(17) $$F_{\mathrm{out}}^{\left(0\right)}=0.5F_{r}+0.5F_{i}.$$

The module thus starts from unbiased Mean Fusion and gradually learns both modality preference and difference-residual strength during optimization.

### 3.4. Selective P3 Fusion Strategy

The final detector concentrates MSDGFM at P3 to exploit high-resolution spatial detail and retains parameter-free Mean Fusion at P4/P5. This allocation directs learned cross-modal interaction to the pyramid level most closely associated with fine vehicle contours in the proposed architecture. Section 4.10 evaluates the placement sensitivity of P3-only, P4-only, P5-only, P3–P4, and P3–P5 configurations on the validation split.

P3 has the highest spatial resolution among the three backbone outputs and preserves more fine-grained vehicle contours, edges, and local spatial layout. These characteristics are particularly relevant to aerial oriented-object detection, where targets are frequently small, densely distributed, and strongly rotated. Applying adaptive difference-guided fusion at this stage allows cross-modal complementary cues to be modeled before substantial spatial compression.

At deeper P4 and P5 levels, the final model uses parameter-free Mean Fusion. Denoting the Mean Fusion operator by ℳ(⋅,⋅), the three cross-modal outputs are

(18) $$F^{3}=\mathrm{MSDGFM}\left(F_{r}^{3},F_{i}^{3}\right),$$

(19) $$F^{4}=M\left(F_{r}^{4},F_{i}^{4}\right),\;F^{5}=M\left(F_{r}^{5},F_{i}^{5}\right).$$

Section 4.10 reports fusion placement using validation-set metrics, while the independent fixed-model comparisons on the Test split assess detection performance.

### 3.5. Multi-Scale Neck and Oriented Detection Head

The fused P3, P4, and P5 features are passed to the YOLO11 PAN/FPN neck. Starting from P5, the top-down path performs nearest-neighbor upsampling and concatenates the result with the fused P4 feature. A C3k2 block produces the intermediate P4 representation. This feature is subsequently upsampled and concatenated with the fused P3 feature, followed by another C3k2 block to form the high-resolution P3 neck output.

The bottom-up path then downsamples the P3 neck feature and combines it with the intermediate P4 representation. A second downsampling stage combines the resulting P4 neck feature with the fused P5 representation. The final three neck outputs contain 64, 128, and 256 channels.

They are jointly supplied to the Ultralytics oriented bounding box head:

{**N**^3^,**N**^4^,**N**^5^} = 𝒩(**F**^3^,**F**^4^,**F**^5^), (20)

**Y** = ℋ*_OBB_*(**N**^3^,**N**^4^,**N**^5^), (21)

where 𝒩(⋅) denotes the PAN/FPN neck and ℋ*_OBB_*(⋅) denotes the OBB prediction head. The detector predicts five categories: car, truck, bus, van, and freight car.

The downstream neck and OBB head remain structurally close to the YOLO11n-OBB baseline. The principal architectural changes are therefore concentrated on two components: independent RGB–IR feature extraction and selective difference-guided cross-modal fusion. This design enables the contribution of MSDGFM to be isolated through controlled ablation experiments.

## 4. Experiments

The evaluation first establishes the performance of single-modal OBB detectors and direct RGB–IR Mean Fusion. It then tests the proposed module against an independently implemented CMAFF-style RGB–IR fusion baseline, uses a controlled 2 × 2 ablation to separate global and local component effects, and examines pyramid placement on the validation split. Three-seed results for the principal models, class-wise performance, computation, and qualitative behavior complete the evidence chain.

### 4.1. Dataset and Preprocessing

The experiments are conducted on DroneVehicle, a paired RGB–infrared aerial vehicle dataset designed for cross-modal vehicle detection [14]. The dataset contains 28,439 RGB–IR image pairs collected from multiple urban scenes covering both daytime and nighttime conditions. Five vehicle categories are used in this study: car, truck, bus, van, and freight car.

The original images have a spatial resolution of 840 × 712 pixels. To remove peripheral regions and maintain a consistent region of interest across the two modalities, each RGB–IR pair is cropped using a fixed rectangle

(*x*_1_,*y*_1_,*x*_2_,*y*_2_) = (100,100,740,612), (22)

resulting in a cropped image size of 640 × 512 pixels. Objects that violate the strict crop-boundary criterion are discarded during preprocessing. Category names are normalized to the five target classes, and polygon annotations are converted into the oriented-bounding-box format required by the detector.

Table 2 lists training (fit), validation (checkpoint/placement), and test (evaluation) splits.

During model training and evaluation, the cropped 640 × 512 images are resized by the Ultralytics letterbox pipeline to an input canvas of 640 × 640. Thus, the crop operation defines the retained scene region, while the network input resolution remains 640 × 640.

The final shared-label Test set contains 115,167 oriented object instances across 8980 image pairs. Among these Test images, 443 contain no target instances after the shared-label filtering procedure.

### 4.2. Shared RGB–IR Label Protocol

To isolate sensing-modality effects, RGB and infrared branches are evaluated against a common supervision target. Because the original DroneVehicle RGB and IR annotations can differ in object count and spatial correspondence, we construct a Shared RGB–IR label protocol that standardizes target count and geometry before training and evaluation.

The experiments use the deterministic Shared RGB–IR label protocol described below. The construction is deterministic and is performed independently for each image pair and each vehicle class. Let *R_c_* and *I_c_* denote the RGB and IR OBB sets of class *c*. Polygon intersection over union (IoU) is computed for every pair in *R_c_* × *I_c_*. Candidate pairs are sorted by IoU in descending order and greedily accepted as a one-to-one match if neither object has already been assigned and

IoU*_poly_*(*r*,*i*) ≥ 0.50. (23)

Objects that remain unmatched are recorded in the RGB-only or IR-only metadata partitions but are excluded from the Shared-label supervision set. For every accepted pair, the class label is unchanged and the RGB polygon is retained as the shared OBB geometry. The same resulting label line is then written to the RGB and IR Shared-label directories. Algorithm 1 summarizes this procedure.
**Algorithm 1.** Shared RGB–IR OBB construction for one paired image.1. Parse the RGB and IR normalized four-point OBB annotations.2. For each class independently, compute polygon IoU for all RGB–IR candidate pairs.3. Sort candidates in descending IoU order.4. Greedily accept unused RGB/IR pairs with polygon IoU ≥0.50, enforcing one-to-one matching.5. For each accepted pair, retain the class and use the RGB polygon as the shared geometry.6. Write the identical shared annotation set for both the RGB and IR images of the pair; exclude unmatched objects from Shared-label training/evaluation.

The Shared RGB–IR label protocol standardizes label count and target geometry across RGB-only, IR-only, and RGB–IR models, providing one consistent supervision geometry for all final comparisons. Verification confirmed identical RGB/IR label files for all 17,990 training, 1469 validation, and 8980 Test pairs. The Test set contains 115,167 shared OBB instances with valid normalized coordinates. This protocol is used for all single-modal baselines, dual-modal ablations, mainstream OBB comparisons, and class-wise analyses.

For the final matching threshold (same-class polygon IoU ≥ 0.50), 363,915 common OBB instances are retained over all splits. Relative to the pre-matching per-modality annotation counts, 27,167 RGB annotations (6.95%) and 65,033 IR annotations (15.16%) are excluded. The split-wise counts are reported in Table 3. Exclusion counts refer to annotations, not to image pairs.

We choose a single geometry convention (the RGB polygon of each successfully matched pair) to prevent discrepancies in target coordinates from confounding RGB-only, IR-only, and fused models. This decision does not imply that RGB annotations are intrinsically superior: IR-only or poorly aligned objects may be removed, and the fixed RGB geometry can favor RGB-localization patterns. Accordingly, all quantitative conclusions refer to common matched objects under this shared-label protocol, not to modality-exclusive targets or unmodified native annotations.

### 4.3. Implementation Details

All experiments are implemented using Python 3.11.15, PyTorch 2.12.1+cu126, and Ultralytics 8.4.37. Training and unified Test evaluation are performed on an NVIDIA GeForce RTX 3060 (NVIDIA Corporation, Santa Clara, CA, USA) with 12 GB of graphics processing unit (GPU) memory.

All models use an input resolution of 640 × 640, a batch size of 8, and 100 training epochs. Automatic mixed precision is disabled throughout the experiments. The number of data-loader workers is set to 0 in the Windows training environment to ensure stable execution. Deterministic training is enabled for the three-seed experiments, using random seeds 0, 1, and 2.

The data-augmentation policy is fixed across the controlled experiments to isolate the effect of multimodal fusion. Translation, scaling, and horizontal flipping are retained, while HSV, Mosaic, MixUp, CutMix, and copy-paste augmentation are disabled. The common settings are summarized in Table 4.

The single-modal mainstream OBB models use the fixed learning-rate configuration established for the single-modal baseline experiments:

*lr*_0_ = 0.001,  *lrf* = 0.01. (24)

This configuration is used for YOLOv8n-OBB, YOLO11n-OBB, and YOLO26n-OBB in the mainstream comparison. Initialization uses the corresponding official Ultralytics OBB checkpoints: yolov8n-obb.pt, yolo11n-obb.pt, and yolo26n-obb.pt. The optional capacity control uses the corresponding s-scale OBB checkpoints analogously.

The dual-modal fusion family uses the following fixed configuration:

*lr*_0_ = 5 × 10^−4^,  *lrf* = 0.1. (25)

The remaining optimizer parameters include momentum 0.937, weight decay 5 × 10^−4^, and a three-epoch warm-up. All repeated dual-modal ablations are initialized from the same dual-stream initialization checkpoint, ensuring identical initialization across the controlled fusion variants.

The learning-rate schedule is fixed within each model family, while the data split, input resolution, epoch count, batch size, augmentation policy, seed set, and Test protocol are shared across the controlled comparisons. The fusion-mechanism conclusions are drawn from within-family RGB–IR ablations using one fixed dual-modal training configuration.

All training, validation, test evaluation, and numerical reporting were executed and checked by the authors. The role of AI-assisted software and manuscript preparation is disclosed in the Acknowledgments.

### 4.4. Baselines and Comparison Models

The comparison contains three complementary groups of models.

**Single-modal baselines.** YOLO11n-OBB is trained separately on RGB and IR images using the shared-label protocol. These two models quantify the independent contribution of each sensing modality.

**Mainstream OBB detectors.** YOLOv8n-OBB, YOLO11n-OBB, and YOLO26n-OBB are trained using the stronger single-modal IR input. All three use the same shared IR labels and the same single-modal training protocol. YOLO11n-OBB (RGB) is retained in the comparison table as a modality reference.

**Dual-modal models.** The principal dual-modal baseline is a simple feature-level Mean Fusion model. After applying the same optional 1 × 1 channel projections used by MSDGFM, it is defined exactly as

**F***_mean_* = 1/2(**F***_r_* + **F***_i_*). (26)

The NoDiff variant retains only the global adaptive modality-allocation branch of MSDGFM. It computes pooled RGB–IR features, predicts two logits, normalizes them with softmax, and outputs

**F***_NoDiff_* = *w_r_***F***_r_* + *w_i_***F***_i_*,  *w_r_* + *w_i_* = 1. (27)

Its final modality-weight layer is zero-initialized, so *w_r_* = *w_i_* = 0.5 at initialization. NoDiff contains no explicit absolute-difference tensor in the output pathway, no local difference gate, and no learnable *γ* residual. Consequently, Mean Fusion → NoDiff isolates learned global modality allocation, whereas NoDiff → full MSDGFM isolates the additional explicit locally gated difference pathway. The fusion-placement variants use the same modules consistently: P3 uses MSDGFM and P4/P5 use Mean Fusion in the final model; P3–P4 uses MSDGFM at P3/P4 and Mean Fusion at P5; P3–P5 uses MSDGFM at all three levels.

In the fixed-P3 component study, Mean Fusion (global off/local off), Global-only (global on/local off), Local-only (global off/local on), and full MSDGFM (global on/local on) share the same dual-stream backbone, initialization, training schedule, and P4/P5 Mean Fusion. Global-only retains the Softmax modality-allocation branch; Local-only retains the absolute difference, local gate, and zero-initialized channel-wise residual but replaces the global weights with equal averaging. A separately adapted CMAFF-style reimplementation [8] is also tested at P3. It is not the original authors’ released full-system implementation; the comparison is specific to the common feature-fusion interface. Previous multi-level NoDiff and MSDGFM variants are retained as auxiliary studies but are not treated as an orthogonal P3-only component control.

A complementary seed-0 capacity-control experiment evaluates YOLOv8s-OBB, YOLO11s-OBB, and YOLO26s-OBB to test whether simply scaling standard single-modal detectors reproduces the accuracy achieved by the proposed multimodal design.

### 4.5. Evaluation Metrics

Detection performance is evaluated using precision, recall, mAP@0.50, and mAP@0.50:0.95.

Precision and recall are defined as

(28) $$P=\frac{\mathrm{TP}}{\mathrm{TP}+\mathrm{FP}},$$

and

(29) $$R=\frac{\mathrm{TP}}{\mathrm{TP}+\mathrm{FN}},$$

where *TP*, *FP*, and *FN* denote the numbers of true-positive, false-positive, and false-negative detections, respectively.

Average precision is computed from the precision–recall curve. *AP*_50_ denotes AP at an IoU threshold of 0.50, while *AP*_50:95_ averages AP over IoU thresholds from 0.50 to 0.95. The dataset-level metrics are obtained by averaging over the five vehicle classes:

(30) $$\mathrm{mAP}=\frac{1}{N_{c}}\sum_{c=1}^{N_{c}}AP_{c},$$

where *N_c_* = 5.

For the unified Test evaluation, inference is performed with a confidence threshold of 0.001, a non-maximum suppression (NMS) IoU threshold of 0.7, and a maximum of 300 detections per image. These settings are fixed for all evaluated models.

The paper reports *mAP*@0.50:0.95 as the stricter primary localization metric because aerial vehicles are densely distributed and arbitrarily oriented, making accurate OBB localization particularly important.

### 4.6. Three-Seed Reproducibility Protocol

The principal models are evaluated using random seeds 0, 1, and 2. For each metric *x*, the manuscript reports the three-seed arithmetic mean

(31) $$\bar{x}=\frac{1}{n}\sum_{i=1}^{n}x_{i},\;n=3,$$

together with the sample standard deviation

(32) $$s=\sqrt{\frac{1}{n-1}\sum_{i=1}^{n}{\left(x_{i}-\bar{x}\right)}^{2}}.$$

Three-seed results are therefore presented in the form

(33) $$\bar{x}\pm s.$$

For controlled model comparisons, paired differences are also computed using the same seed index. A result is described as directionally stable only when the improvement has the same sign across all three paired seeds and the mean gain is not dominated by a single run.

Three seeds are used to quantify reproducibility and sensitivity to initialization.

### 4.7. Class-Wise Evaluation

In addition to overall mAP, AP is reported separately for car, truck, bus, van, and freight car. This analysis is used to determine whether the overall improvement is concentrated in the dominant car category or extends to more difficult classes.

### 4.8. Efficiency Benchmark

Model complexity is characterized using parameter count, giga floating-point operations (GFLOPs), model-forward latency, frames per second (FPS), and peak GPU memory.

Runtime efficiency is measured on the same NVIDIA GeForce RTX 3060 using:

1. batch size = 1;

2. 32-bit floating-point (FP32) inference;

3. input size = 640 × 640;

4. 50 CUDA warm-up iterations;

5. 300 timed forward iterations.

The reported latency corresponds to model-forward time, with image loading and validation-pipeline overhead excluded. FPS is calculated as

(34) $$\mathrm{FPS}=\frac{1000}{t},$$

where *t* is the mean model-forward latency in milliseconds.

The efficiency benchmark characterizes the resource profile associated with the reported detection accuracy on the specified hardware.

### 4.9. Main Results Against Mainstream OBB Detectors

**Figure 3:**
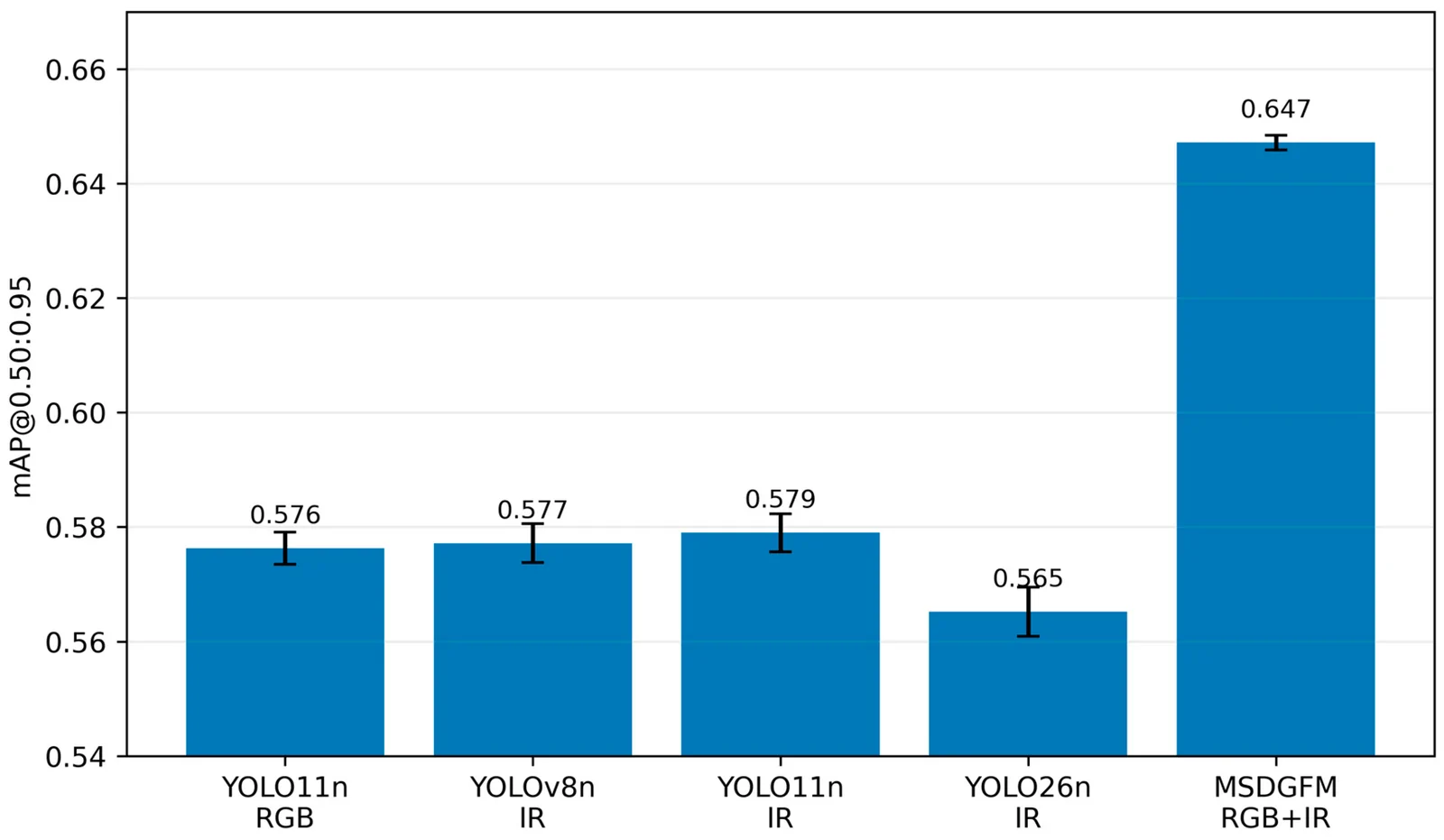
Comparison of mAP@0.50:0.95 between the proposed method and mainstream nano-scale OBB detectors under the unified Test protocol. Error bars indicate the sample standard deviation over three random seeds.

Figure 3 summarizes the three-seed mAP@0.50:0.95 comparison, while Table 5 reports the corresponding precision, recall, mAP@0.50, and mAP@0.50:0.95 values.

Table 5 shows the end-to-end detection advantage of the proposed RGB–IR system under the unified Test protocol. Among the evaluated single-modal detectors, YOLO11n-OBB (IR) is the strongest baseline, reaching 0.7465 ± 0.0042 mAP@0.50 and 0.5790 ± 0.0033 mAP@0.50:0.95. MSDGFM-YOLO11n-OBB (P3) raises these metrics to 0.7853 ± 0.0027 and 0.6472 ± 0.0013, corresponding to gains of 3.88 and 6.82 percentage points.

The gain is highly consistent across repeated training: the proposed model exceeds YOLO11n-OBB (IR) in mAP@0.50:0.95 by +0.0658, +0.0688, and +0.0698 for seeds 0, 1, and 2. Precision and recall also increase to 0.7531 ± 0.0021 and 0.7716 ± 0.0014, showing that the improvement is not restricted to a single operating characteristic.

The comparisons distinguish complementary sensing from targeted fusion refinement. Across three seeds, YOLO11n-OBB (IR) achieves 0.5790 mAP@0.50:0.95, paired RGB–IR Mean Fusion reaches 0.6389 (+5.99 percentage points), and MSDGFM-P3 reaches 0.6472 (a further +0.83 percentage points over Mean Fusion). Their corresponding mAP@0.50 values are 0.7465, 0.7773, and 0.7853. The improvement over a shared dual-stream Mean Fusion baseline identifies the additional benefit associated with the MSDGFM mechanism.

### 4.10. Controlled Fusion Mechanisms, Existing-Method Comparison, and Validation-Only Placement

To isolate the two MSDGFM branches without changing fusion placement, the 2 × 2 study uses the same P3-only fusion site, Mean Fusion at P4/P5, and the same seed-0 training/evaluation protocol throughout (Table 6). It independently switches global modality allocation and the local absolute-difference residual on or off. The original three-level NoDiff ablation remains an auxiliary comparison but is not used to infer the P3 local-branch contribution.

Under the fixed P3 configuration, Global-only and Local-only improve mAP@0.50:0.95 over Mean Fusion by 0.79 and 0.29 percentage points, respectively. Combining the two branches yields 0.6484, the highest seed-0 value in the 2 × 2 study: +0.98 points relative to Mean Fusion and +0.19 points relative to Global-only. The branch effects are reported as controlled single-seed AP differences, without inferring statistical interaction from this experiment.

Table 7 provides a direct method-family comparison with an independent, CMAFF-style reimplementation based on the published common-/differential-attention concept [8]. CMAFF is inserted only at P3; both models retain Mean Fusion at P4/P5. Shared backbone, matched labels, optimizer settings, initialization checkpoint, 100-epoch budget, and seed 0 are held fixed. This adaptation is not an author-released implementation of the complete CMAFF detector.

Within the matched seed-0 fusion interface, MSDGFM achieves 0.6484 mAP@0.50:0.95, compared with 0.6460 for the CMAFF-style adaptation, a difference of +0.24 percentage points. Table 7 also reports precision and recall for both implementations. This comparison evaluates the two feature-fusion designs in a common dual-stream detector rather than comparing complete independently published detection systems.

Table 8 reports seed-0 Validation-only placement sensitivity under the shared 100-epoch protocol. P3 was fixed in the original study, whose placement comparisons used Test results; the supplementary Validation study characterizes sensitivity rather than retrospectively justifying the original choice. Final Test comparisons remain separate.

The validation mAP@0.50:0.95 values are closely grouped: P4-only reaches 0.66113, P3-only 0.65961, and P3–P5 0.66056, with P5-only and P3–P4 also included in Table 8. P4-only is 0.152 percentage points above P3-only on this split. The study characterizes placement sensitivity on Validation, while fixed-model detection comparisons are reported on the separate Test split.

Across three seeds (Table 9), the frozen P3 MSDGFM detector reaches 0.6472 ± 0.0013 mAP@0.50:0.95, compared with 0.6389 ± 0.0004 for direct Mean Fusion; the three paired improvements are +0.0098, +0.0087, and +0.0064. These multi-seed means are reported separately from the single-seed CMAFF-style comparison and the 2 × 2 component controls.

### 4.11. Gains Across Vehicle Categories

**Figure 4:**
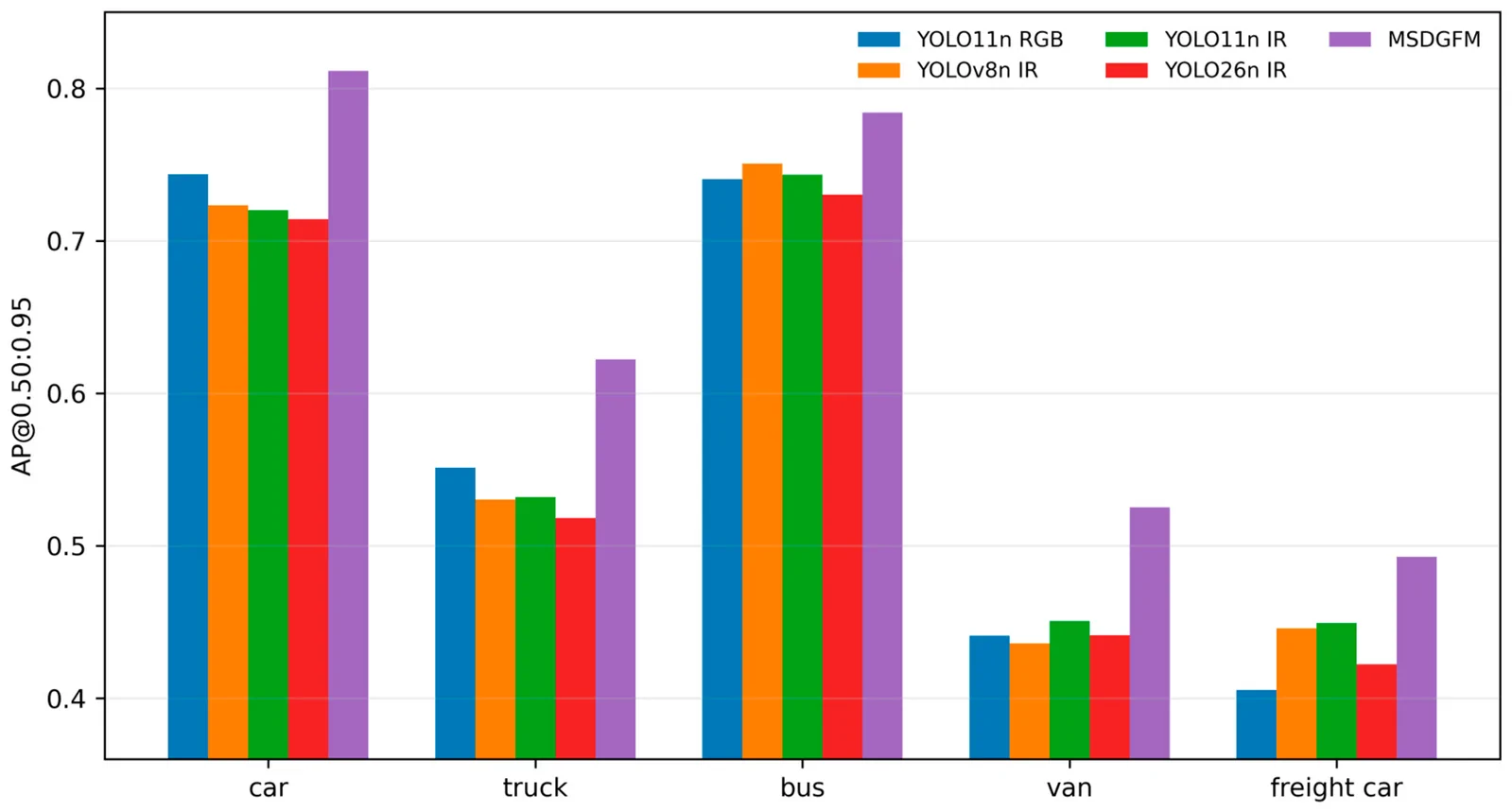
Class-wise AP@0.50:0.95 comparison on the shared-label Test set for car, truck, bus, van, and freight car.

Figure 4 visualizes class-wise AP@0.50:0.95, and Table 10 lists the corresponding values relative to the strongest evaluated single-modal reference for each class.

The benefit of RGB–IR fusion extends across the complete five-class label space. Relative to the best evaluated nano-scale single-modal baseline for each class, MSDGFM-YOLO11n-OBB improves AP@0.50:0.95 for car, truck, bus, van, and freight car. The gains are especially pronounced for van (+0.0747) and truck (+0.0709), while car, bus, and freight car also improve by +0.0678, +0.0336, and +0.0433, respectively.

This distribution of gains strengthens the central claim: the proposed detector improves categories with different visual characteristics, with the largest improvements appearing in classes where single-modal discrimination is more demanding.

### 4.12. Accuracy–Computation Profile and Capacity Control

**Figure 5:**
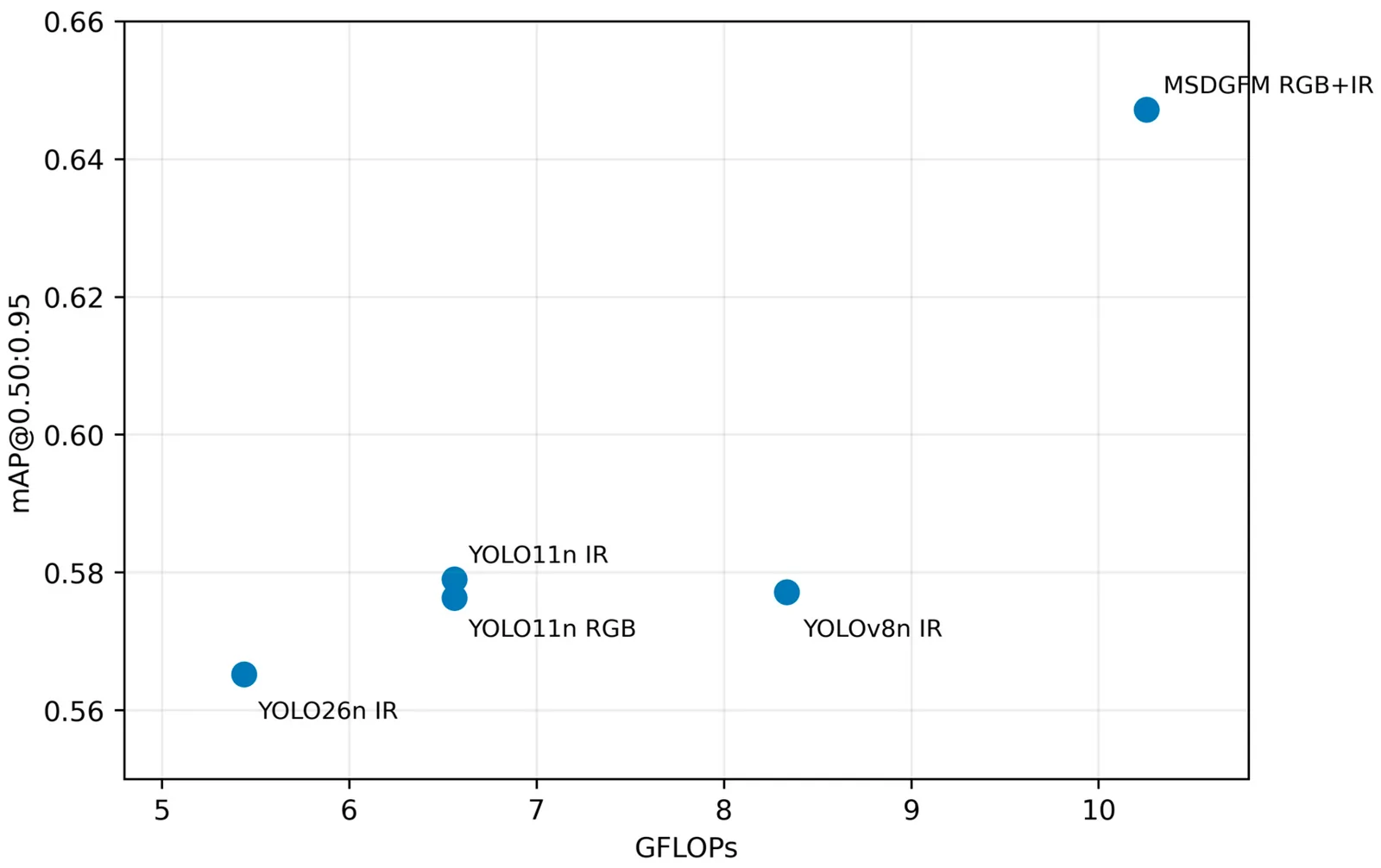
Accuracy–computation profile of the main model families. Accuracy is the three-seed mean mAP@0.50:0.95, and computation is measured in GFLOPs.

Figure 5 shows the accuracy–computation profile of the main model families, and Table 11 reports their corresponding accuracy and runtime/resource measurements.

The proposed detector uses 4.069 M parameters and 10.26 GFLOPs, while maintaining 144.6 FPS model-forward throughput on the RTX 3060 in batch-1 FP32 inference. This resource profile accompanies a 6.82-point improvement in mAP@0.50:0.95 over YOLO11n-OBB (IR), the strongest evaluated nano-scale single-modal baseline.

The seed-0 capacity-control comparison (Table 12) uses YOLO11s-OBB (IR), which has 9.701 M parameters and 22.29 GFLOPs, and achieves 0.6052 mAP@0.50:0.95. The proposed model reaches 0.6484 with 58.1% fewer parameters and 54.0% fewer FLOPs. This comparison assesses accuracy relative to model capacity under the stated single-modal and multimodal training configurations; it does not establish a same-modality architecture-only gain.

### 4.13. Adaptive Modality Behavior and Qualitative Results

**Figure 6:**
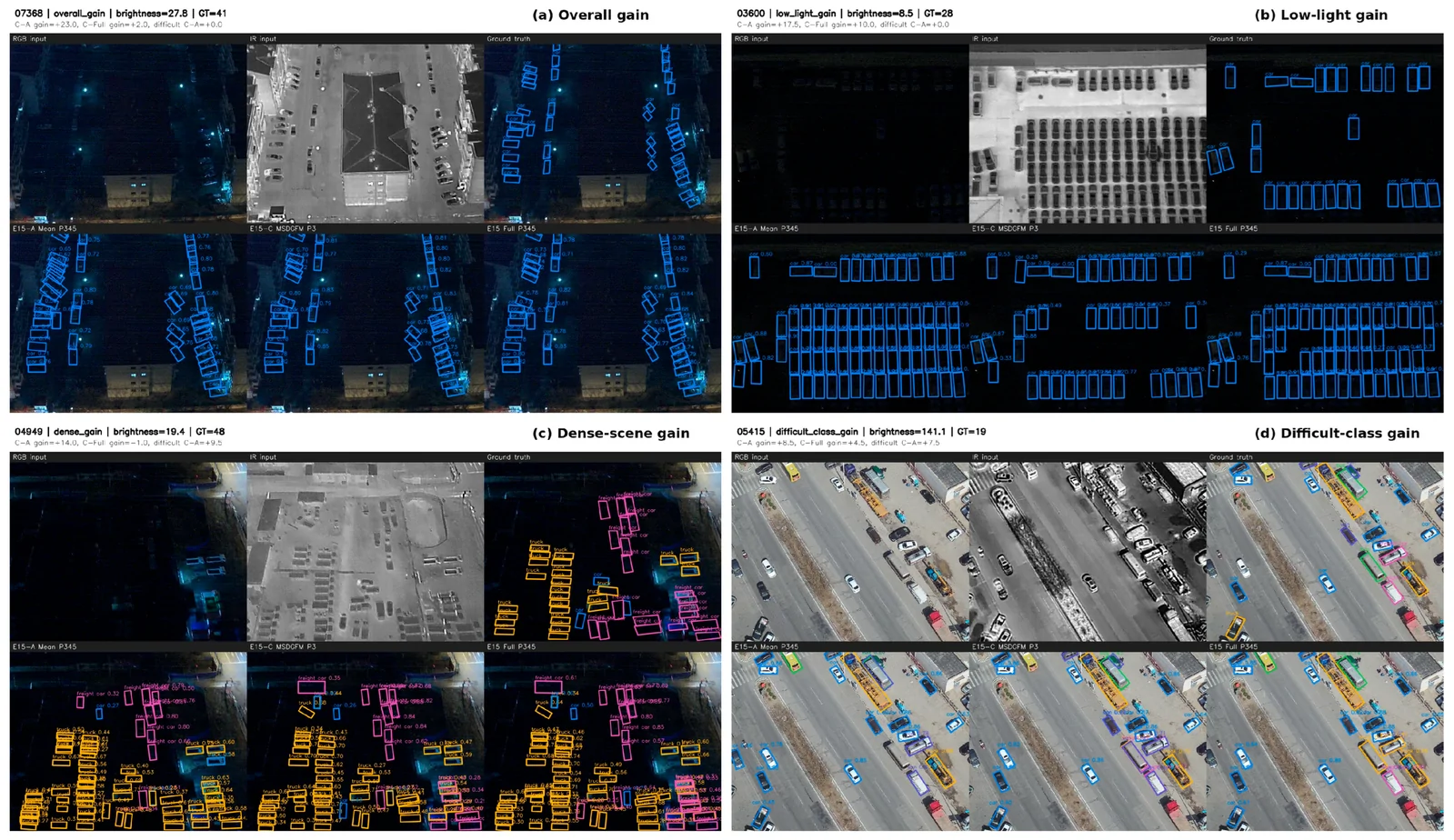
Representative qualitative detection comparisons for overall-gain, low-light, dense-scene, and difficult-class cases. Each panel shows RGB input, IR input, ground truth, Mean Fusion, MSDGFM P3, and MSDGFM P3–P5. Colored rotated boxes indicate ground-truth or predicted oriented bounding boxes, with outline colors distinguishing vehicle categories; text annotations on predictions indicate class labels and confidence scores.

**Figure 7:**
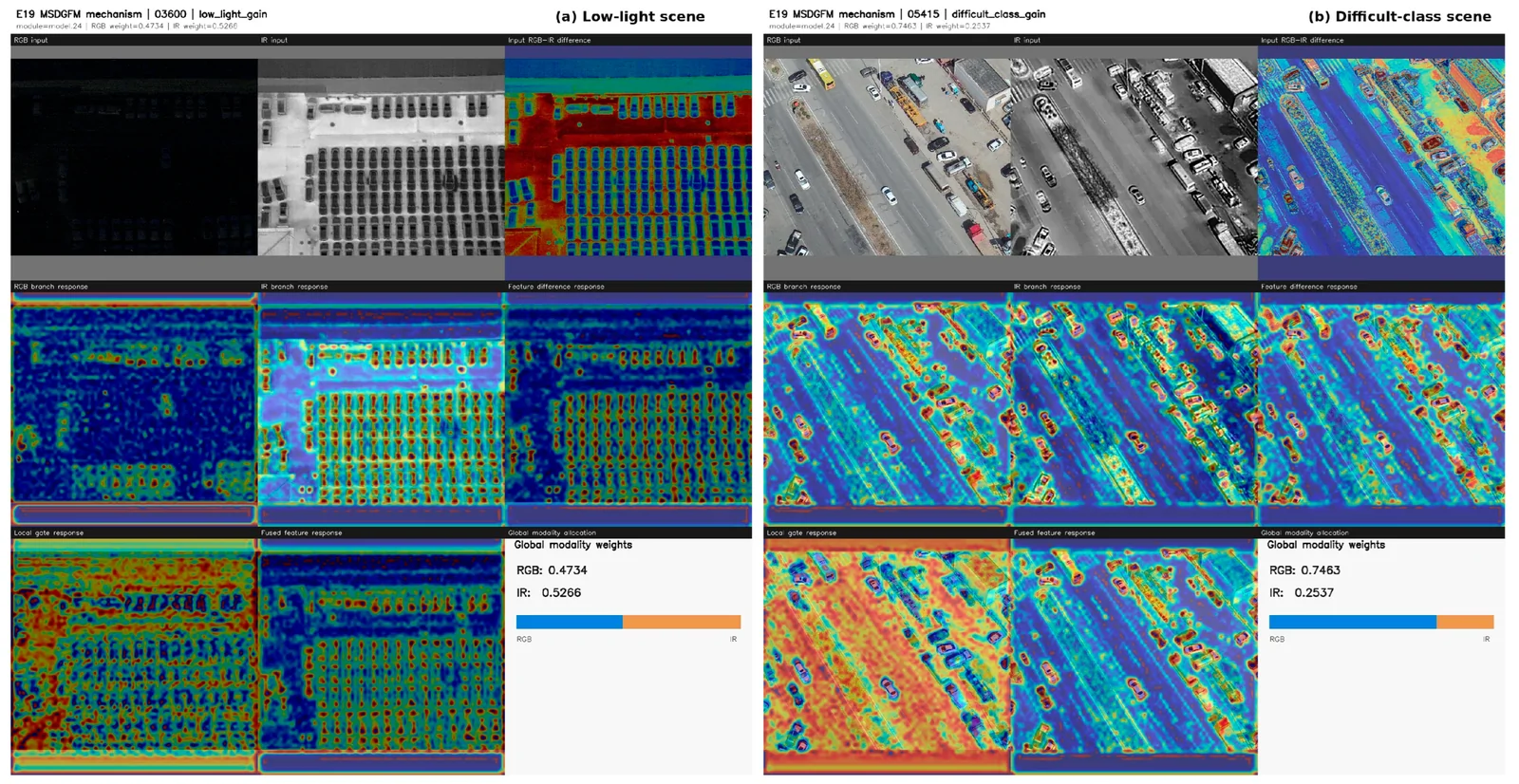
Representative MSDGFM mechanism visualizations for (**a**) a low-light scene (**left**) and (**b**) a difficult-class scene (**right**). Heatmaps are independently normalized for visualization; global RGB/IR allocations are shown in the bottom-right panels. In the independently normalized response maps, warmer colors denote relatively higher displayed responses and cooler colors denote lower responses; the bar plots show the sample-specific RGB/IR allocation weights.

Figure 6 and Figure 7 present representative qualitative detection results and internal MSDGFM visualizations, respectively.

Figure 6 shows representative overall-gain, low-light, dense-vehicle, and difficult-class scenes. Across these cases, MSDGFM P3 recovers targets or suppresses false detections more effectively than direct Mean Fusion while preserving the same RGB–IR input pair and downstream OBB formulation.

Figure 7 provides a direct view of the learned fusion behavior. In a representative low-light scene, the global RGB/IR allocation is approximately 0.473/0.527, giving slightly greater weight to infrared information. In a representative difficult-class scene, the allocation shifts to approximately 0.746/0.254, placing greater emphasis on RGB appearance cues. The local gate and fused-response maps further show that this allocation is complemented by spatially varying difference injection, rather than by a fixed global preference alone.

Together, these examples connect the quantitative gain to the intended mechanism: MSDGFM changes how the two modalities contribute as scene content changes, while the P3 placement preserves high-resolution information for oriented localization.

### 4.14. Discussion

The controlled results locate the value of MSDGFM within the RGB–IR fusion process. Paired sensing first raises the three-seed mean mAP@0.50:0.95 from 0.5790 with YOLO11n-OBB (IR) to 0.6389 with Mean Fusion; refining that shared dual-stream representation with MSDGFM-P3 raises it to 0.6472. The positive paired gains across seeds 0, 1, and 2 are supported by fixed-P3 global/local controls, which separately quantify the two branches. A seed-0 CMAFF-style adaptation provides a further same-framework reference for the resulting feature-fusion behavior.

The architecture links these gains to a concrete design choice: learned modality allocation handles sample-dependent RGB/IR contributions, while the spatial gate introduces cross-modal difference information at the high-resolution P3 stage. Positive class-wise changes across car, truck, bus, van, and freight car show that the improvement is distributed across the five evaluated vehicle categories. With 4.069 M parameters and 10.26 GFLOPs, the detector provides a compact dual-modal system; the seed-0 capacity control also records 4.32 percentage points higher mAP@0.50:0.95 than YOLO11s-OBB (IR) with fewer parameters and FLOPs, as a system-level accuracy–capacity comparison.

The evaluation concerns commonly matched vehicles under the DroneVehicle Shared-label protocol: class-wise polygon matches use IoU ≥ 0.50, and the RGB polygon supplies the common annotation geometry. This setup holds target geometry fixed across RGB-only, IR-only, and paired models, while unmatched or modality-exclusive objects fall outside the evaluated set and RGB-based geometry can influence modality-specific localization comparisons. The conclusions therefore apply to matched-target aerial RGB–IR OBB detection; evaluation under native annotations or other datasets addresses a distinct setting.

## 5. Conclusions

MSDGFM-YOLO11n-OBB provides a selective fusion strategy for aerial RGB–IR oriented vehicle detection. Independent modality backbones feed a P3 module that combines sample-dependent Softmax allocation with a spatially gated absolute-difference residual, while P4/P5 retain parameter-free Mean Fusion. Exact Mean Fusion initialization provides a controlled starting point for learning both modality preference and local complementary corrections.

The final MSDGFM-YOLO11n-OBB (P3) reaches 0.7853 ± 0.0027 mAP@0.50 and 0.6472 ± 0.0013 mAP@0.50:0.95 over three random seeds, improving the strongest evaluated single-modal nano-scale OBB baseline by 3.88 and 6.82 percentage points. Under the same RGB–IR dual-stream family, it improves direct Mean Fusion on all three repeated seeds, and the gain extends across all five vehicle categories. In the seed-0 capacity control, it also exceeds YOLO11s-OBB (IR) by 4.32 percentage points in mAP@0.50:0.95 while using 58.1% fewer parameters and 54.0% fewer FLOPs.

Controlled global/local ablations identify the roles of the two fusion branches, and validation-set placement analysis characterizes selective high-resolution fusion alongside deeper-level alternatives. Together with the consistent three-seed gains over a matched Mean Fusion baseline, these findings demonstrate the value of targeted cross-modal refinement in a compact aerial RGB–IR OBB detector under the defined Shared-label evaluation protocol.

## Figures and Tables

**Table 1:** Representative multispectral methods and their cross-modal design characteristics.

Method	Detection Setting	Cross-Modal Design
Sun et al./DroneVehicle [14]	Aerial RGB–IR vehicle detection	Uncertainty-aware fusion; paired aerial benchmark
Yuan et al./TSRA [15]	Aerial RGB–IR vehicle detection	Translation–scale–rotation alignment
DGE-YOLO [16]	UAV multispectral detection	Dual-stream feature aggregation
Cross-modal edge-enhanced detector [17]	UAV multispectral detection	Edge-guided complementary enhancement
MO R-CNN [18]	Multispectral oriented detection	Heterogeneous multimodal representation for OBB
DMM [19]	Multispectral oriented detection	Disparity-aware Mamba fusion
GEM-YOLO [21]	RGB–thermal detection	Global modality gating; multi-level adaptive fusion
GMD-YOLO [22]	YOLO11 dual-modality detection	Gradient-aware fusion within a multi-component detector
TFFB [26]	Visible–infrared small-target detection	Cross-Transformer fusion at P3/P4/P5
MCF-YOLO [27]	UAV RGB–IR detection	Hierarchical high-resolution/deep fusion
AMSRDet [28]	DroneVehicle UAV detection	Global fusion and adaptive multi-scale weighting
MDCFT-HSM [23]	UAV RGB–T small-object detection	Protected-modality residual injection at multiple stages
SIM-PCSR [30]	DroneVehicle UAV RGB–IR HBB detection	P3-centered selective interaction and refinement
MSDGFM-YOLO11n-OBB (ours)	Aerial RGB–IR oriented vehicle detection	Sample-level allocation + gated difference residual; P3 adaptive fusion, P4/P5 Mean Fusion

**Table 2:** Dataset split used in this study.

Split	RGB–IR Pairs
Train	17,990
Validation	1469
Test	8980
Total	28,439

**Table 3:** Final Shared-label matching audit (IoU ≥ 0.50; annotation counts before/after matching).

Split	RGB Original	IR Original	Shared	RGB Excluded (%)	IR Excluded (%)
Train	247,649	270,933	230,568	17,081 (6.90%)	40,365 (14.90%)
Val	19,430	21,053	18,180	1250 (6.43%)	2873 (13.65%)
Test	124,003	136,962	115,167	8836 (7.13%)	21,795 (15.91%)
Total	391,082	428,948	363,915	27,167 (6.95%)	65,033 (15.16%)

**Table 4:** Common training configurations.

Item	Setting
Input resolution	640 × 640
Epochs	100
Batch size	8
Optimizer	AdamW
Seeds	0, 1, 2 (main); 0 (supplementary)
AMP	Disabled
Deterministic mode	Enabled
Translation	0.1
Scale	0.5
Horizontal flip	0.5
HSV augmentation	0
Mosaic	0
MixUp	0
CutMix	0
Copy-paste	0

**Table 5:** Mainstream OBB comparison over three random seeds.

Model	Input	Precision	Recall	mAP@0.50	mAP@0.50:0.95
YOLO11n-OBB	RGB	0.7230 ± 0.0103	0.7182 ± 0.0036	0.7323 ± 0.0039	0.5763 ± 0.0028
YOLOv8n-OBB	IR	0.7065 ± 0.0060	0.7356 ± 0.0033	0.7409 ± 0.0038	0.5772 ± 0.0034
YOLO11n-OBB	IR	0.7142 ± 0.0035	0.7408 ± 0.0045	0.7465 ± 0.0042	0.5790 ± 0.0033
YOLO26n-OBB	IR	0.7046 ± 0.0095	0.7255 ± 0.0048	0.7293 ± 0.0043	0.5652 ± 0.0043
MSDGFM-YOLO11n-OBB (P3)	RGB + IR	0.7531 ± 0.0021	0.7716 ± 0.0014	0.7853 ± 0.0027	0.6472 ± 0.0013

**Table 6:** Controlled P3 2 × 2 fusion-component ablation on the shared-label Test split (seed 0).

P3 Variant	Global/Local	Precision	Recall	mAP@0.50	mAP@0.50:0.95
Mean Fusion	0/0	0.7497	0.7608	0.7769	0.6386
Global-only	1/0	0.7534	0.7694	0.7843	0.6465
Local-only	0/1	0.7440	0.7636	0.7797	0.6415
MSDGFM (ours)	1/1	0.7513	0.7732	0.7879	0.6484

**Table 7:** Representative RGB–IR feature-fusion comparison under the unified Test protocol (seed 0).

P3 Fusion Variant	P4/P5	Precision	Recall	mAP@0.50	mAP@0.50:0.95
Mean Fusion	Mean/Mean	0.7497	0.7608	0.7769	0.6386
CMAFF-style [8]	Mean/Mean	0.7426	0.7794	0.7850	0.6460
MSDGFM (ours)	Mean/Mean	0.7513	0.7732	0.7879	0.6484

**Table 8:** Validation-only MSDGFM fusion-placement sensitivity (seed 0; mAP@0.50:0.95 used for checkpoint selection).

Adaptive Levels	Remaining Levels	Best Val Epoch	Val mAP@0.50	Val mAP@0.50:0.95
P3	P4/P5 Mean	31	0.80028	0.65961
P4	P3/P5 Mean	23	0.80217	0.66113
P5	P3/P4 Mean	23	0.79539	0.65610
P3–P4	P5 Mean	22	0.79983	0.65807
P3–P5	None	36	0.79839	0.66056

**Table 9:** Three-seed reproducibility of the principal fusion variants (mean ± sample standard deviation).

Variant	Fusion Placement	mAP@0.50	mAP@0.50:0.95
Mean Fusion	P3–P5	0.7773 ± 0.0016	0.6389 ± 0.0004
MSDGFM (ours)	P3	0.7853 ± 0.0027	0.6472 ± 0.0013
MSDGFM	P3–P5	0.7822 ± 0.0024	0.6440 ± 0.0021

**Table 10:** Class-wise AP@0.50:0.95 compared with the best evaluated nano-scale single-modal baseline for each class.

Class	Proposed AP@0.50:0.95	Best Single-Modal AP@0.50:0.95	Gain
car	0.8115 ± 0.0004	0.7437 (YOLO11n-OBB)	+0.0678
truck	0.6222 ± 0.0054	0.5513 (YOLO11n-OBB)	+0.0709
bus	0.7842 ± 0.0024	0.7506 (YOLOv8n-OBB)	+0.0336
van	0.5253 ± 0.0027	0.4505 (YOLO11n-OBB)	+0.0747
freight car	0.4926 ± 0.0033	0.4493 (YOLO11n-OBB)	+0.0433

**Table 11:** Accuracy and resource profile of the main model families.

Model	Input	mAP@0.50:0.95	Params (M)	GFLOPs	Latency (ms)	FPS	Peak GPU Memory (MB)
YOLO11n-OBB	RGB	0.5763 ± 0.0028	2.655	6.56	4.259	234.8	86.2
YOLOv8n-OBB	IR	0.5772 ± 0.0034	3.078	8.33	3.469	288.3	89.4
YOLO11n-OBB	IR	0.5790 ± 0.0033	2.655	6.56	4.250	235.3	86.2
YOLO26n-OBB	IR	0.5652 ± 0.0043	2.447	5.44	5.308	188.4	83.9
MSDGFM-YOLO11n-OBB (P3)	RGB + IR	0.6472 ± 0.0013	4.069	10.26	6.917	144.6	137.7

**Table 12:** Seed-0 model-capacity control.

Model	Input	Params (M)	GFLOPs	mAP@0.50	mAP@0.50:0.95
YOLOv8s-OBB	IR	11.414	29.43	0.7698	0.6038
YOLO11s-OBB	IR	9.701	22.29	0.7735	0.6052
YOLO26s-OBB	IR	9.753	21.51	0.7618	0.5975
MSDGFM-YOLO11n-OBB (P3)	RGB + IR	4.069	10.26	0.7879	0.6484

## Data Availability

The DroneVehicle dataset used in this study is publicly available from the original dataset providers [14] at https://github.com/VisDrone/DroneVehicle (accessed on 10 September 2026). The processed Shared-label annotations, model configurations, experimental configurations, and evaluation scripts generated in this study are available from the corresponding author upon reasonable request.

## References

[1] Xia G.-S., Bai X., Ding J., Zhu Z., Belongie S., Luo J., Datcu M., Pelillo M., Zhang L. (2018). DOTA: A Large-Scale Dataset for Object Detection in Aerial Images. Proceedings of the IEEE Conference on Computer Vision and Pattern Recognition (CVPR).

[2] Ding J., Xue N., Long Y., Xia G.-S., Lu Q. (2019). Learning RoI Transformer for Oriented Object Detection in Aerial Images. Proceedings of the IEEE/CVF Conference on Computer Vision and Pattern Recognition (CVPR).

[3] Han J., Ding J., Xue N., Xia G.-S. (2021). ReDet: A Rotation-Equivariant Detector for Aerial Object Detection. Proceedings of the IEEE/CVF Conference on Computer Vision and Pattern Recognition (CVPR).

[4] Xie X., Cheng G., Wang J., Yao X., Han J. (2021). Oriented R-CNN for Object Detection. Proceedings of the IEEE/CVF International Conference on Computer Vision (ICCV).

[5] Li W., Chen Y., Hu K., Zhu J. (2022). Oriented RepPoints for Aerial Object Detection. Proceedings of the IEEE/CVF Conference on Computer Vision and Pattern Recognition (CVPR).

[6] Zhou K., Chen L., Cao X. (2020). Improving Multispectral Pedestrian Detection by Addressing Modality Imbalance Problems. Computer Vision—ECCV 2020.

[7] Zhang H., Fromont E., Lefèvre S., Avignon B. (2021). Guided Attentive Feature Fusion for Multispectral Pedestrian Detection. Proceedings of the IEEE/CVF Winter Conference on Applications of Computer Vision (WACV).

[8] Fang Q., Wang Z. (2022). Cross-Modality Attentive Feature Fusion for Object Detection in Multispectral Remote Sensing Imagery. Pattern Recognit..

[9] Fang Q., Han D., Wang Z. (2021). Cross-Modality Fusion Transformer for Multispectral Object Detection. arXiv.

[10] Shen J., Chen Y., Liu Y., Zuo X., Fan H., Yang W. (2024). ICAFusion: Iterative Cross-Attention Guided Feature Fusion for Multispectral Object Detection. Pattern Recognit..

[11] Deevi S.A., Lee C., Gan L., Nagesh S., Pandey G., Chung S.-J. (2024). RGB-X Object Detection via Scene-Specific Fusion Modules. Proceedings of the IEEE/CVF Winter Conference on Applications of Computer Vision (WACV).

[12] Jang J., Park C., Kim H., Lee J., Paik J. (2025). Multispectral Object Detection Enhanced by Cross-Modal Information Complementary and Cosine Similarity Channel Resampling Modules. Proceedings of the IEEE/CVF Winter Conference on Applications of Computer Vision (WACV).

[13] Zhu H., Dong W., Yang L., Li H., Yang Y., Ren Y., Zhu Q., Feng Z., Li C., Lin S. (2025). WaveMamba: Wavelet-Driven Mamba Fusion for RGB-Infrared Object Detection. Proceedings of the IEEE/CVF International Conference on Computer Vision (ICCV).

[14] Sun Y., Cao B., Zhu P., Hu Q. (2022). Drone-Based RGB-Infrared Cross-Modality Vehicle Detection Via Uncertainty-Aware Learning. IEEE Trans. Circuits Syst. Video Technol..

[15] Yuan M., Wang Y., Wei X. (2022). Translation, Scale and Rotation: Cross-Modal Alignment Meets RGB-Infrared Vehicle Detection. Computer Vision—ECCV 2022.

[16] Lv K., Xiao Z., Ren H., Li X., Lan P. (2026). DGE-YOLO: Dual-Branch Gathering and Attention for Efficient Accurate UAV Object Detection. Appl. Sci..

[17] Li G., Ren G., Wang J., Zhi M., Yu Z., Jiang B., Guan H., Guo Q. (2026). Cross-Modal Edge-Enhanced Detector for UAV-Based Multispectral Object Detection. Sci. Rep..

[18] Wang L., Jin B., Huang F., Chen L., Wang Z., He X., Chen H. (2026). Multispectral-Oriented R-CNN for Object Detection in Remote Sensing Images. J. Appl. Remote Sens..

[19] Zhou M., Li T., Qiao C., Xie D., Wang G., Ruan N., Mei L., Yang Y., Shen H.T. (2025). DMM: Disparity-Guided Multispectral Mamba for Oriented Object Detection in Remote Sensing. IEEE Trans. Geosci. Remote Sens..

[20] Yuan M., Wei X. (2024). C^2^Former: Calibrated and Complementary Transformer for RGB-Infrared Object Detection. IEEE Trans. Geosci. Remote Sens..

[21] Wang L., Bao Z., Lu D. (2026). GEM-YOLO: A Lightweight and Real-Time RGBT Object Detector with Gated Multimodal Fusion. Sensors.

[22] Lin Z., Wang X., Guo Y. (2026). GMD-YOLO: A Dual-Modality Framework with Multi-Scale Enhancement and Adaptive Fusion for PV Fault Detection. Sensors.

[23] Long T., Wang Y., Yuan H., Zhang Q., Ma H., Zhou R., Wang Z., Wang F. (2026). MDCFT-HSM: Modality-Shift-Aware RGB-T Small-Object Detection for Low-Altitude UAV Remote Sensing. Sensors.

[24] Tang D., Yang W., Fu Q., Xuan J. (2026). Intelligent Small Object Detection in Aerial Images via Cross-Modal Attention Interaction. Acta Aeronaut. Astronaut. Sin..

[25] Li H., Wang Z., Zhou L., Lei S. (2026). A Study on a Multimodal Fusion-Based Method for Small Object Detection in UAV Aerial Images. Comput. Technol. Dev..

[26] Dong Y., Xie C., Zhang C., Hu Q., Han X., Xu C. (2026). Small-Target Detection via Fusion of Visible and Infrared Image Features. Sensors.

[27] Yang X., Yang M., Xie X. (2026). MCF-YOLO: Consistency-Guided Cross-Modal Attention for Small-Object RGB-IR Detection. Sensors.

[28] Yan Z., Li Y. (2026). AMSRDet: An Adaptive Multi-Scale UAV Infrared-Visible Remote Sensing Vehicle Detection Network. Sensors.

[29] Zhao X., Zhang H., Li C., Wang K., Zhang Z. (2025). DVIF-Net: A Small-Target Detection Network for UAV Aerial Images Based on Visible and Infrared Fusion. Remote Sens..

[30] He J., Yang Y., Li J. (2026). SIM-PCSR: Key-Layer Complementary Enhancement for UAV RGB-IR Small-Object Detection. Sensors.

[31] Su S., Li Z., Lu R., Li L., Yang X. (2026). Frequency Purification and Interactive Fusion for UAV-Based VIS-IR Object Detection. Acta Aeronaut. Astronaut. Sin..

